# Neuropsychiatric symptoms in mild cognitive impairment and early Alzheimer's disease: Clinical pattern and diagnostic implications

**DOI:** 10.3934/Neuroscience.2025032

**Published:** 2025-12-18

**Authors:** Donna de Levante Raphael

**Affiliations:** Alzheimer's Foundation of America, Research Department, 322 8th Avenue, Floor 16, New York, NY 10001, USA

**Keywords:** Alzheimer's disease, depression, anxiety, neuropsychiatric symptoms of dementia, NPS, dementia, MCI, behavior, apathy

## Abstract

**Background:**

Alzheimer's disease (AD) and mild cognitive impairment (MCI) are widely recognized for their hallmark cognitive deficits, typically characterized by progressive cognitive deterioration. However, neuropsychiatric symptoms (NPS), including depression, apathy, anxiety, irritability, and sleep disturbances, are increasingly prevalent in the early stages of these conditions and significantly influence the disease trajectory and patient outcomes. Importantly, neuropsychiatric symptoms often precede overt memory loss by several years, with subtle mood and behavioral disturbances serving as early pre-diagnostic markers of an underlying Alzheimer's pathology. Their presence complicates the diagnosis, accelerates the disease progression, and intensifies the caregiver burden. However, distinguishing NPS arising from neurodegeneration and primary psychiatric disorders remains a profound diagnostic challenge, thus delaying timely intervention and obscuring early disease recognition.

**Objective:**

This structured narrative review examines the diagnostic complexities, clinical impact, and current management of NPS in early-stage Alzheimer's disease (AD) and Mild Cognitive Impairment (MCI), alongside the biological underpinnings, clinical relevance, diagnostic challenges, and treatment perspectives. We argue that understanding and managing NPS is essential to improve the clinical outcomes, reduce the caregiver burden, and guide therapeutic innovation.

**Methods:**

A structured narrative review of peer-reviewed studies published between 2012 and 2025 was conducted using PubMed, MEDLINE, Scopus, PsycINFO, Google Scholar, and CINAHL. The included studies investigated NPS prevalence, neurobiological correlations, and management strategies in individuals with AD or MCI.

**Findings:**

NPS affects up to 80% of individuals with early AD or MCI, often preceding cognitive decline. The current management strategies heavily rely on non-pharmacological interventions such as caregiver support, behavioral activation, and structured routines, while pharmacological options remain limited by modest efficacy and safety concerns.

**Discussion:**

Advancing knowledge of NPS and their association with cognitive decline is critical to establish more precise diagnostic criteria and to inform personalized therapeutic approaches. Future research should emphasize biomarker-driven diagnostics and the development of novel, targeted interventions that simultaneously address cognitive and neuropsychiatric domains to optimize outcomes for patients and caregivers. This study contributes to the field by reframing NPS as potential early biomarkers in the trajectory of MCI and dementia progression.

## 1. Introduction

Alzheimer's disease (AD) and mild cognitive impairment (MCI) are classically defined progressive deficits in cognition [1]. However, an expanding body of evidence emphasizes the prominence and clinical significance of neuropsychiatric symptoms (NPS) in these conditions, particularly during the early stages of disease. Rather than being secondary or comorbid phenomena, NPS are now understood to be integral to the neurodegenerative process itself, with important implications for diagnosis, prognosis, and management [2],[3].

NPS encompass a broad spectrum of behavioral and psychological disturbances, including apathy, depression, anxiety, agitation, disinhibition, hallucinations, delusions, irritability, sleep disruptions, and psychosis [4],[5]. Epidemiological studies suggest that up to 80% of individuals with early-stage AD or MCI present with at least one clinically relevant NPS, and many experience multiple symptoms concurrently [4],[6]. These manifestations not only exacerbate cognitive and functional decline but also increase the caregiver burden, reduce the quality of life (QoL), and complicate clinical assessments [4],[7].

While the timeline is not yet definitive, emerging evidence indicates that certain NPS may precede measurable cognitive impairment and act as early clinical markers of underlying neurodegenerative changes [8],[9].

Neuroimaging studies have identified structural and functional abnormalities, particularly within the medical temporal lobe and fronto-limbic circuits, that correlate with specific NPS profiles [10],[11]. In parallel, the dysregulation of key neurotransmitter systems (including serotonin, dopamine, and glutamate), along with genetic vulnerabilities such as APOE ε4 status, have been implicated in the pathophysiology of these symptoms [12],[13].

Clinically, the presence of NPS in individuals with MCI has been associated with an increased risk of progression to AD, more rapid cognitive decline, and poorer overall outcomes [14],[15].

Moreover, these symptoms can obscure the diagnostic clarity, delay therapeutic interventions, and limit the effectiveness of cognitive screening tools. Despite their significance, the current treatment options for NPS in early AD and MCI remain suboptimal. Non-pharmacological interventions such as cognitive-behavioral therapy and caregiver-based strategies offer modest benefits. These approaches, such as psychoeducation, behavioral therapy, and environmental modifications, have demonstrated some benefit, particularly when integrated into personalized care plans that involve caregivers [16].

Pharmacological treatments frequently yield inconsistent outcomes and carry risks of adverse events. These interventions, including antidepressants, antipsychotics, and anxiolytics, have shown an inconsistent efficacy in this population and carry risks of adverse effects, especially in older adults [16].

Despite their clinical significance, NPS remain under-addressed in standard diagnostic and treatment paradigms. Traditional cognitive screening tools often overlook behavioral symptoms, and there is a lack of consensus on standardized approaches for NPS assessment in early-stage disease [16]. Moreover, treatment options are limited.

In sum, NPS are prevalent, impactful, and biologically grounded manifestations of early AD and MCI. They provide a crucial clinical window into the underlying neurodegenerative process and offer potential opportunities for early detection and intervention. A better understanding of the mechanisms, trajectories, and treatment responses of NPS will be vital to redefine the diagnostic criteria, develop effective therapies, and improve the patient and caregiver outcomes.

## 2. Materials and methods

### 2.1. Study design and approach

This study followed a structured narrative review design aimed at synthesizing peer-reviewed evidence published between January 2012 and February 2025 on the prevalence, diagnostic complexities, neurobiology, and management of NPS in early-stage AD and MCI. The review adhered to key elements of the Preferred Reporting Items for Systematic Reviews and Meta-Analyses (PRISMA) framework to ensure methodological rigor, though a meta-analysis was not conducted due to heterogeneity across study designs and outcome measures.

### 2.2. Search strategy

A comprehensive search was conducted across six major electronic databases: PubMed, MEDLINE, Scopus, PsycINFO, CINAHL, and Google Scholar. The search covered publications from 2012 to 2025. Keywords and Boolean operators were used in combinations to capture the relevant literature. The primary search string included the following: “Neuropsychiatric symptoms” OR “behavioral and psychological symptoms of dementia” OR “NPS” OR “BPSD”, “Alzheimer's disease” OR “early Alzheimer's” OR “mild cognitive impairment” OR “MCI”, “depression” OR “apathy” OR “anxiety” OR “agitation” OR “psychosis” OR “irritability” OR “sleep disturbance” and “early detection” OR “diagnosis” OR “preclinical” OR “biomarker” OR “intervention”.

Manual searches of the reference lists of key review articles and recent systematic reviews were performed to identify additional studies not captured by the electronic databases. Only peer-reviewed journal articles written in English were included.

### 2.3. Selection criteria

#### 2.3.1. Inclusion criteria

Studies were included if they met the following criteria: examined individuals with early-stage AD, prodromal AD, or MCI with documented neuropsychiatric symptoms; reported data on the prevalence, clinical presentation, neurobiological correlates, or management of NPS; included quantitative, qualitative, or mixed-methods designs; utilized validated diagnostic tools (e.g., NPI, CSDD, GDS, DSM-5, NIA-AA criteria); and were published in peer-reviewed journals between 2012 and 2025.

#### 2.3.2. Exclusion criteria

Studies were excluded if they met the following criteria: exclusively focused on moderate to severe AD, vascular, frontotemporal, or Lewy body dementias; did not differentiate between early-stage and late-stage AD or lacked clarity on disease staging; were conference abstracts, editorials, commentaries, or case reports without original data; solely focused on pharmacological efficacy without characterizing NPS phenotypes; and were non-English or not peer-reviewed.

### 2.4. Data collection and extraction

Titles and abstracts of all retrieved studies were independently screened by a reviewer to determine relevance. Full texts of potentially eligible studies were reviewed in detail. Data were extracted using a structured data extraction form developed for this review, which captured the following domains to include study characteristics: author(s), year, country, study design, sample size, and population characteristics; clinical parameters such as type, prevalence, and severity of NPS; diagnostic criteria for AD or MCI; assessment tools: instruments used to evaluate NPS; management strategies of pharmacological and non-pharmacological interventions, outcomes, and adverse effects; and key findings such as main results, limitations, and implications for early detection and clinical management. Each study was rated as high, moderate, or low quality based on the clarity of methodology, representativeness of sample, use of validated instruments, and robustness of analysis. Inter-rater reliability for quality assessment was calculated using Cohen's kappa (κ = 0.82), which indicated a strong agreement.

### 2.5. Data synthesis and analysis

Given the heterogeneity across study designs, measurement tools, and reported outcomes, a narrative synthesis was employed. Studies were grouped according to five primary analytical themes: Prevalence and Phenotypic Patterns of NPS in Early AD and MCI, Neurobiological and Genetic Underpinnings, Diagnostic Complexities and Differential Challenges, Impact on Disease Progression, Caregiver Burden, and QoL and Treatment and Management Strategies (Pharmacologic and Non-Pharmacologic).

Each section integrates quantitative and qualitative findings to provide a cohesive understanding of how NPS manifest, evolve, and influence disease outcomes in early AD and MCI.

As this review utilized published, publicly available data, ethical approval was not required. All included studies were assumed to have received appropriate institutional ethical clearances.

## 3. Prevalence and impact of NPS

### 3.1. Overview of NPS

NPS encompass a range of behavioral and psychological disturbances, including apathy, depression, anxiety, irritability, sleep disturbances, and psychosis. Studies indicate that up to 80% of individuals with early-stage AD or MCI exhibit at least one significant NPS, with many experiencing multiple concurrent symptoms [17]. These symptoms are associated with accelerated cognitive decline, an increased caregiver burden, and a reduced QoL.

### 3.2. Neurobiological underpinnings

The pathophysiology of NPS in early AD and MCI involves complex interactions between structural and functional brain changes, neurotransmitter dysregulation, and genetic factors. Neuroimaging studies have identified correlations between specific symptoms and abnormalities in brain regions such as the medial temporal lobe and fronto-limbic circuits [18]. Additionally, the dysregulation of neurotransmitter systems, including serotonin, dopamine, and glutamate, has been implicated in the emergence of NPS. Genetic factors, such as the presence of the APOE ε4 allele, may also influence the development of these symptoms [19].

### 3.3. Recent studies and findings

Over the past five years, research has significantly advanced understanding of NPS in early AD and MCI. The findings indicate that more than half of individuals with MCI exhibit at least one NPS, with depression, apathy, irritability, and sleep disturbances being the most common [20]. Neuroimaging consistently demonstrates that these symptoms are associated with structural changes, particularly reduced volumes in the orbitofrontal and posterior cingulate cortices, thus underscoring their neuroanatomical basis [20]. Furthermore, the prevalence and severity of NPS appear to increase in parallel with cognitive decline, with individuals diagnosed with AD showing substantially higher rates of depression and apathy compared to those with amnestic MCI [16]. Collectively, these findings emphasize that NPS are not incidental but intrinsic to the disease process, and their presence may provide valuable insights into both the prognosis and the underlying neuropathology.

### 3.4. Prevalence chart

**Figure 1.**
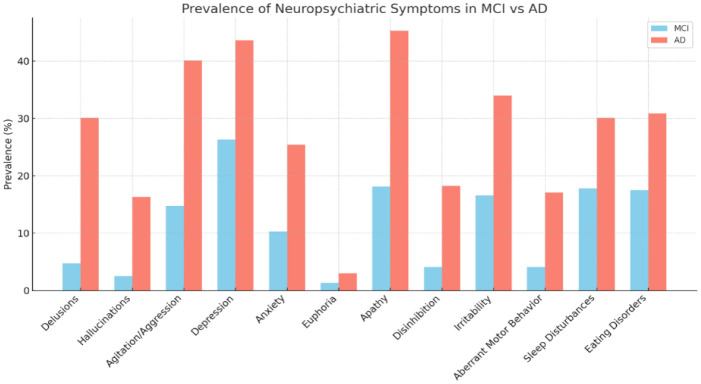
Prevalence of individual NPS in individuals with MCI and AD.

The following chart summarizes the prevalence of NPS in individuals with MCI and AD, based on data from the Cardiovascular Health Study [20] (Figure 1).

## 4. The impact of neuropsychiatric symptoms on disease progression in early-stage Alzheimer's disease and mild cognitive impairment

NPS are increasingly recognized as not only prevalent in early AD and MCI, but also as key modulators of disease progression. These symptoms are now understood to have a profound influence on the trajectory of cognitive and functional decline, thereby contributing to a more rapid evolution of disease, an increased caregiver burden, and poorer clinical outcomes [21].

### 4.1. Acceleration of cognitive decline

Multiple longitudinal studies have demonstrated that the presence of NPS in individuals with MCI significantly increases the risk of progression to AD. For instance, apathy and depression are consistently identified as independent predictors of faster cognitive deterioration. A meta-analysis found that individuals with MCI and comorbid depression had a 2- to 3-fold increased risk of converting to AD within 2–3 years [22]. Similarly, apathy, which is associated with frontal-subcortical circuit dysfunction, has been shown to correlate with reduced executive function and diminished motivation to engage in cognitively stimulating activities, both of which are linked to accelerated neurodegeneration.

Moreover, NPS may synergistically interact with underlying neuropathological processes. For example, individuals that exhibit early anxiety or depression show greater amyloid-β accumulation, hippocampal atrophy, and tau pathology, thus suggesting that NPS may not simply be reactive symptoms, but instead reflect early disease mechanisms that are driving both behavioral and cognitive changes.

### 4.2. Functional impairment and decline in activities of daily living

Beyond cognition, NPS exert a detrimental effect on the functional capacity. Symptoms such as agitation, irritability, and disinhibition interfere with the instrumental activities of daily living (IADLs), including medication adherence, financial management, and social engagement. Functional decline is an important clinical marker of disease advancement, and its onset is often hastened in the presence of NPS [16].

Apathy and depression are associated with decreased goal-directed behavior, poor engagement in rehabilitation or cognitive interventions, and social withdrawal [16]. These effects lead to reduced mental and physical stimulation, which further exacerbates cognitive decline in a vicious cycle. The presence of even mild behavioral symptoms in MCI is predictive of an increased and rapid loss of independence and an earlier transition to moderate or severe stages of dementia.

### 4.3. Neurobiological pathways and disease mechanisms

The mechanistic pathways linking NPS to disease progression are increasingly being delineated through advances in neuroimaging and biomarker research. Emerging evidence indicates that NPS correspond to distinct patterns of neurodegeneration and synaptic dysfunction [23]. For instance, apathy has been associated with structural atrophy in the anterior cingulate and orbitofrontal cortices [24]. Similarly, depressive and anxious symptomatology have been linked to a reduced hippocampal volume alongside alterations in limbic and paralimbic networks [25]. Psychotic manifestations, including hallucinations and delusions, demonstrate correlations with elevated tau deposition and pronounced cortical thinning within temporal and parietal regions [24].

### 4.4. Impact on diagnostic clarity and intervention timing

NPS have the potential to obscure the clinical presentation of cognitive decline, thereby delaying an appropriate diagnostic evaluation [26]. For example, depressive symptomatology in older adults is often misattributed to a primary psychiatric disorder, which can contribute to the underdiagnosis or misdiagnosis of MCI or early AD. Similarly, early behavioral changes may be misinterpreted, thus postponing the use of neuroimaging or biomarker-based assessments and limiting opportunities for timely interventions.

Cognitive assessments serve as fundamental tools to quantify subtle changes in memory and executive functions, thus enabling the early detection of neurodegenerative processes and the evaluation of disease progression. Additionally, these assessments provide objective, standardized measures of other domains essential for detecting, characterizing, and tracking cognitive decline across the AD continuum. NPS can compromise the validity of cognitive assessments. Mood disturbances, apathy, and related features may reduce test engagement or effort, thus resulting in artificially diminished performances that do not accurately reflect the underlying cognitive capacity [27],[28]. This diagnostic complexity introduces significant challenges for clinical decision-making and may delay the initiation of both disease-modifying and symptomatic treatments.

### 4.5. Increased caregiver burden and institutionalization

NPS can significantly impact the caregiver burden and the overall QoL [29]. Behavioral manifestations such as aggression, wandering, and sleep-wake disturbances represent leading contributors to caregiver burnout and are robust predictors of earlier institutional placements [30]. Following institutionalization, individuals who present with prominent NPS are more likely to require psychotropic pharmacotherapy, exhibit increased risk of falls, and demonstrate accelerated physical and cognitive decline.

The extensive burden imposed by NPS on both family caregiving systems and broader healthcare infrastructures highlights the critical need for early recognition, systematic monitoring, and the proactive management of these symptoms across the trajectory of the disease.

### 4.6. Therapeutic challenges and opportunities

Despite their clinical significance, the management of NPS in early-stage AD and MCI remains suboptimal. Pharmacologic treatments (e.g., Selective Serotonin Reuptake Inhibitors (SSRIs), antipsychotics, cholinesterase inhibitors) offer limited and often inconsistent benefits, and carry significant risks such as falls, sedation, and cerebrovascular events. Non-pharmacologic approaches—such as structured activity programs, caregiver training, and environmental modifications—have demonstrated some efficacy but are underutilized.

There is growing interest in developing targeted interventions that address the specific neurobiological underpinnings of NPS, including novel neuromodulation techniques (e.g., transcranial magnetic stimulation), anti-inflammatory agents, and personalized behavioral therapies based on neuroimaging profiles [31].

## 5. Impact of neuropsychiatric symptoms on quality of life

NPS in early AD and MCI are among the most distressing manifestations of neurodegenerative decline, profoundly impacting both the affected individuals and their caregivers. Unlike core cognitive symptoms that gradually progress, NPS often abruptly emerge, unpredictably fluctuate, and can significantly undermine the QoL from the earliest stages of disease [32].

### 5.1. Patient distress and loss of autonomy

Apathy and social withdrawal are common in early AD and MCI and often diminish engagement in meaningful activities, thus leading to a sense of isolation, loss of purpose, and a reduced subjective well-being [33].

Furthermore, symptoms such as irritability, disinhibition, and sleep disturbances interfere with daily routines and disrupt interpersonal relationships, thus increasing the emotional burden on patients who may still possess partial insight into their behavioral changes. The cumulative effect of NPS is a reduced ability to participate in social, recreational, and even basic functional activities, all of which are vital contributors to the perceived QoL.

### 5.2. Functional decline and dependence

NPS are strongly associated with functional impairment and frequently precede measurable declines in IADLs, including financial management, medication adherence, and the maintenance of social relationships. Symptoms such as apathy and depression can significantly diminish the motivation to engage in self-care and rehabilitative activities, thereby accelerating dependence on caregivers [34],[35]. As their functional abilities deteriorate, individuals with dementia become increasingly reliant on informal or formal care providers to accomplish daily tasks. This progressive loss of independence constitutes a critical inflection point in the disease trajectory and is often accompanied by emotional distress, frustration, and diminished self-esteem factors that collectively contribute to a reduced overall QoL.

### 5.3. Increased caregiver burden

The presence of NPS markedly amplifies caregiver stress and has been shown to negatively influence the patient outcomes. Behavioral manifestations such as aggression, hallucinations, repetitive vocalizations, and nocturnal wandering are particularly challenging to manage within home settings and are consistently identified as the primary precipitants of caregiver burnout. Compared to caregivers of individuals exhibiting predominantly cognitive decline, those caring for patients with prominent NPS report higher levels of depression, anxiety, and physical health problems [29]. The behavioral episodes often disrupt the caregiver's employment, social engagement, and personal health maintenance, thus compounding the caregiving burden.

### 5.4. Institutionalization and healthcare costs

NPS are a major driver of early and prolonged institutionalization. NPS and disorders are often observed in AD and affect up to 97% of all AD-affected individuals during their AD journey [36]. Behavioral symptoms, especially those that pose safety risks (e.g., aggression, delusions, wandering), often lead families to seek out long-term care facilities when home-based support becomes unsustainable. Once institutionalized, patients with prominent NPS are more likely to receive psychotropic medications, experience more frequent hospitalizations, and have poorer long-term outcomes.

The economic implications of institutional care are significant. Institutional care costs are substantially higher than community-based care, and patients with NPS tend to require more intensive supervision, staffing, and medical management. The cost of NPS in dementia care can range from $15,000 to approximately $85,000 per dementia patient on an annual basis. This high cost can exacerbate the socioeconomic burden of AD and related dementia [37]. Therefore, NPS not only diminish the individual's QoL but also impose considerable strain on public and private healthcare systems.

## 6. Diversity and disparities in neuropsychiatric symptoms of early dementia

Understanding the role of diversity in the presentation, diagnosis, and treatment of NPS in AD and MCI is essential for equitable and personalized dementia care. While NPS are common across populations, their expression, recognition, and management are shaped by a complex interplay of biological, cultural, social, and systemic factors that significantly vary across diverse groups.

### 6.1. Racial and ethnic disparities in NPS prevalence and recognition

Research suggests that the prevalence and types of NPS may differ across racial and ethnic groups, although findings remain mixed due to methodological inconsistencies and the underrepresentation of minorities in clinical studies [38],[39]. For example, Black and Hispanic/Latino individuals with dementia are often reported to have higher rates of delusions, hallucinations, and agitation compared to non-Hispanic Whites [38],[39], while White patients may more frequently report or be diagnosed with apathy and depression, possibly reflecting differences in symptom reporting or clinician interpretation [38].

These disparities may be influenced by cultural belief systems regarding emotional expression, stigma around mental illness, and barriers to access to diagnostic and psychiatric services. In many minority populations, behavioral symptoms may be normalized, underreported, or attributed to spiritual or familial stressors rather than pathophysiological changes, which lead to underdiagnoses or delayed interventions [40]–[42].

### 6.2. Gender differences in NPS expression and impact

Gender also plays a significant role in the manifestation and burden of NPS. Women with MCI or early AD are more likely to experience depression and anxiety, while men may exhibit higher rates of agitation, aggression, and disinhibition [41],[43]. These differences may reflect underlying neurobiological mechanisms such as inflammation, hormonal influences, socialization patterns, and caregiving dynamics [43],[44]. Furthermore, female caregivers who comprise the majority of unpaid dementia caregivers are disproportionately affected by the emotional and physical toll of managing NPS. Moreover, their experiences may differ from male caregivers in terms of the reported burden, coping strategies, and access to support.

### 6.3. Socioeconomic and educational influences

Lower socioeconomic status (SES) is associated with a greater burden of dementia-related symptoms, including NPS. Individuals from disadvantaged backgrounds are less likely to receive early diagnostic assessments or specialist care and are more often managed in under-resourced community settings [45]. This may result in a prolonged exposure to untreated behavioral symptoms, thus increasing the caregiver stress and the risk of institutionalization. Additionally, educational attainment can influence how NPS are perceived and managed. Higher education is generally associated with better health literacy and a greater engagement with cognitive health, which may facilitate earlier recognition of subtle behavioral changes [46]. Conversely, lower education levels may contribute to diagnostic overshadowing, where NPS are misattributed to aging, stress, or cultural factors.

### 6.4. Cultural and linguistic barriers

Cultural context plays a critical role in shaping both the expression and interpretation of NPS across diverse populations [47]. In certain cultural groups, emotional or psychological distress may present as somatization, with behavioral and affective disturbances conveyed through physical complaints rather than verbalized emotional states. Likewise, cognitive behavioral manifestations, such as hallucinations, paranoia, or delusional thinking, are often interpreted through religious or spiritual belief systems, influencing not only symptom meaning but also patterns of help-seeking and the adherence to prescribed treatments. Linguistic differences and language barriers can further exacerbate diagnostic challenges, particularly when standardized screening tools are not culturally adapted, translated, or validated for the target population [47]. These limitations, compounded by clinician bias and cultural misinterpretation, can lead to inaccurate symptom reporting, diagnostic disparities, and the under recognition of behavioral and psychological symptoms of dementia in minoritized communities.

### 6.5. Implications for research and practice

The existing literature on NPS in AD and MCI is often constrained by the underrepresentation of diverse populations in clinical and research settings. Longitudinal cohorts and clinical trials have predominantly enrolled non-Hispanic White, highly educated, and urban-dwelling participants, thus resulting in a study population that does not reflect the demographic heterogeneity of those affected by AD and MCI. This lack of diversity substantially restricts the generalizability of findings and hinders the development of diagnostic tools and therapeutic interventions that are culturally sensitive and equitable.

### 6.6. Research priorities

To address these critical gaps, future investigations should implement inclusive recruitment strategies. These strategies refer to deliberate, equity-centered approaches designed to ensure the participation of diverse populations in research studies that actively engage underrepresented populations in research, employ culturally validated instruments for the assessment of NPS, examine the sociocultural determinants that shape symptom expression, help-seeking behaviors, and access to care, and advance community-based participatory research approaches to foster trust, engagement, and long-term partnerships with diverse communities.

### 6.7. Clinical implications

From a clinical perspective, healthcare providers should be adequately trained to recognize the ways in which a cultural background influences the manifestation and interpretation of NPS. Clinicians should employ culturally informed communications, as well as the capacity to tailor diagnostic and therapeutic strategies to the social and cultural strategies to improve communication with individual patients and their families.

### 6.8. Summary of disparities

Table 1 provides an overview of key disparities in the expression and impact of NPS in early AD and MCI. The table highlights the influence of race, ethnicity, gender, socioeconomic status, and cultural factors on symptom recognition, caregiver burden, and access to care.

## 7. Diagnostics of neuropsychiatric symptoms in early Alzheimer's disease and mild cognitive impairment

NPS are increasingly recognized as integral components of the clinical spectrum of AD and MCI, especially in the early stages [33],[48]. However, diagnosing NPS in these conditions presents distinct challenges due to their heterogeneous nature, overlap with other psychiatric disorders, and the potential for misinterpretation of behavioral changes as normal age-related phenomena. The accurate identification and diagnostic classification of NPS are critical not only for providing optimal care but also for improving prognostication and guiding therapeutic interventions [33],[48].

This section outlines the complexities of diagnosing NPS in AD and MCI, thereby discussing the currently available tools and methodologies, challenges in clinical practice, and emerging trends in diagnostic approaches.

### 7.1. Diagnostic challenges in NPS

#### 7.1.1. Heterogeneity of symptoms

NPS in early-stage AD and MCI encompass a wide range of psychological and behavioral changes. Each symptom may significantly vary in terms of intensity, frequency, and impact across individuals, thus complicating the diagnostic process [33]. Furthermore, these symptoms often occur in isolation or in combination, which makes it challenging to distinguish them from one another or from other neuropsychiatric conditions.

The challenge is further compounded by the fact that NPS can appear at any stage of cognitive decline, often preceding measurable cognitive deficits or appearing in conjunction with them [33],[48]. For example, apathy and depression may emerge long before significant memory impairment, thus making these symptoms difficult to attribute to Alzheimer's pathology in the absence of a formal cognitive diagnosis.

**Table 1.** Disparities in expression and impact of NPS in early AD and MCI.

**Factor**	**Disparity**	**Implications for Diagnosis and Treatment**
**Racial/Ethnic Differences**	Black, and Hispanic/Latino patients may experience higher rates of delusions, hallucinations, and agitation compared to non-Hispanic Whites.	These populations may have distinct neurobiological profiles and symptom expressions, influencing both recognition and management of NPS.
	White patients may more frequently present with apathy, depression, and anxiety.	Cultural differences in emotional expression and stigma could affect symptom reporting and diagnostic accuracy.
**Gender Differences**	Women with AD or MCI are more likely to experience depression and anxiety.	Differences in symptom expression may impact treatment decisions, as interventions for depression and anxiety may differ from those for agitation or aggression.
	Men may exhibit higher rates of agitation, aggression, and disinhibition.	Gender differences in NPS can influence caregiver stress and the type of care required.
**Socioeconomic Status (SES)**	Lower SES is associated with higher prevalence of NPS and greater caregiver burden.	Limited access to healthcare and diagnostic services may delay detection and appropriate management of NPS, leading to worsened outcomes.
	Individuals with low SES may be more prone to institutionalization earlier due to unmanaged NPS.	The high cost of healthcare, lack of community resources, and the impact on caregiver resources may drive institutionalization at earlier stages.
**Education Level**	Lower education levels are associated with greater difficulty in recognizing and managing NPS.	Lack of health literacy can result in poor awareness of symptoms, delays in diagnosis, and reduced engagement with available interventions.
**Cultural Factors**	In certain cultures, somatization of NPS may occur, where emotional symptoms manifest as physical complaints.	Cultural norms can influence the way symptoms are expressed and perceived, possibly leading to misdiagnosis or delayed intervention.
	Religious or spiritual interpretations of symptoms may be more common in some cultural groups.	Cultural interpretations may delay or impede medical diagnosis, as symptoms may be seen as spiritual or supernatural phenomena.
**Language Barriers**	Non-English speaking patients may face challenges in understanding diagnostic tools and participating in therapy.	Linguistic differences can lead to misunderstandings in symptom reporting and diagnostic assessments, compromising treatment efficacy.

#### 7.1.2. Overlap with other psychiatric disorders

NPS, particularly depression and anxiety, often co-occur with primary psychiatric disorders such as major depressive disorder (MDD), generalized anxiety disorder (GAD), and bipolar disorder, thus complicating the differentiation between primary psychiatric conditions and those stemming from neurodegenerative processes [49]. For instance, individuals with MCI or early AD may present with depression that is qualitatively different from that observed in late-life depression. The etiology of this depression may be tied to the underlying neurodegenerative processes in the brain rather than typical psychosocial stressors or genetic predispositions [49].

The misdiagnosis of primary psychiatric disorders may lead to inappropriate treatments, as medications and therapies effective for mood disorders may not address the neurobiological causes of NPS in dementia [33].

#### 7.1.3. Cognitive decline as a confounding factor

The cognitive decline inherent to AD and MCI presents another layer of complexity in diagnosing NPS. As cognitive impairments progress, the ability of patients to accurately report or self-identify psychological symptoms diminishes [50],[51]. This can lead to underreporting or the misinterpretation of NPS by the patient, which may cause clinicians to heavily rely on caregiver reports, thus often leading to biases in symptom attribution.

In some cases, caregivers may attribute behavioral changes to the patient's cognitive decline or aging, thereby failing to recognize them as distinct neuropsychiatric symptoms that require specific management. This is especially true for less overt symptoms such as apathy or irritability, which may not be immediately alarming but significantly impair the QoL.

## 8. Tools for diagnosing NPS in early AD and MCI

### 8.1. Clinical interviews and standardized questionnaires

The most common diagnostic strategies to assess NPS rely on structured clinical interviews and standardized questionnaires that evaluate both cognitive and behavioral domains. These instruments typically contain sections devoted to specific neuropsychiatric features, including mood disturbances, anxiety, sleep disorders, and psychotic symptoms, thus allowing for a more comprehensive evaluation of the patient's clinical profile [52].

Among the most widely utilized tools is the Neuropsychiatric Inventory (NPI), which has become a standard measure in both research and clinical practice. The NPI evaluates twelve domains, including delusions, hallucinations, agitation, depression, anxiety, and apathy, and provides information about the frequency and severity of symptoms. Despite its widespread adoption, the NPI predominantly relies on caregiver reports, which can limit its accuracy, particularly when symptoms are subtle, intermittent, or less observable by others.

Another frequently employed measure is the Geriatric Depression Scale (GDS), a self-report tool for screening depressive symptoms in older adults, including those with AD and MCI. The GDS has been validated for use in individuals with cognitive impairments and is valued for its ease of administration and specificity in detecting depression. However, its reliability diminishes in cases of advanced cognitive decline, as patients may have difficulty comprehending or accurately responding to items [52].

The Cornell Scale for Depression in Dementia (CSDD) was specifically developed to assess depression in individuals with dementia [53]. In contrast to the GDS, it integrates information from both patients and evaluates mood, behavioral features, and physical symptoms. This dual-source approach makes the CSDD particularly well-suited for populations with significant cognitive impairments [53]. Nevertheless, challenges remain in distinguishing depression as a primary psychiatric disorder from depressive symptoms that arise as secondary consequences of neurodegenerative changes.

Collectively, these instruments represent essential tools for the systematic evaluation of NPS, yet each carries inherent limitations. Their effective application requires a careful clinical interpretation, particularly in differentiating between psychiatric and neurodegenerative etiologies, and underscores the continued need for the development of more sensitive and contextually valid assessment methods.

### 8.2. Neuroimaging and biomarkers

Advances in neuroimaging and biomarker research have substantially enhanced diagnostic precision in AD and MCI. However, despite their widespread use in detecting and monitoring cognitive decline, these modalities are not yet routinely employed to evaluate NPS. Structural imaging modalities, such as magnetic resonance imaging (MRI) and positron emission tomography (PET), are particularly valuable to detect neurodegenerative changes that align with both cognitive deterioration and behavioral alterations [54],[55].

Functional magnetic resonance imaging (fMRI) has been especially important in revealing disruptions in brain network connectivity, particularly within the frontal lobes and limbic structures. These regions play central roles in emotional regulation and behavioral control, and dysfunction in these circuits has been associated with irritability, apathy, agitation, and other NPS [56]. Similarly, PET imaging with amyloid or tau tracers, which are primarily employed to detect hallmark AD pathology, has begun to demonstrate relevance for NPS. Emerging evidence suggests that tau accumulation and amyloid burden correlate not only with cognitive decline but also with affective and psychotic features, including depression and hallucinations [57],[58]. These findings underscore the potential of molecular imaging to refine the diagnostic accuracy in MCI and early AD, particularly by linking pathological burden with symptom-specific presentations.

Beyond neuroimaging, biomarker investigations have increasingly emphasized the role of inflammation in the pathogenesis of NPS. Peripheral inflammatory markers, including cytokines and C-reactive protein (CRP), as well as cerebrospinal fluid (CSF) indices, have been implicated in the onset and severity of neuropsychiatric manifestations in dementia [59],[60]. Although these markers remain at an experimental stage and have not yet been validated for clinical practice, they hold promise for elucidating mechanistic pathways that connect systemic immune dysregulation to the behavioral and psychological symptoms of dementia.

Despite these advances, significant limitations remain. Neuroimaging techniques such as fMRI and PET are costly, not universally accessible, and often limited to research settings, which restricts their utility in routine clinical practice. Moreover, while associations between pathology and NPS have been documented, causal relationships remain difficult to establish due to the complex, multifactorial etiology of these symptoms [14]. Similarly, biomarker studies of inflammation are complicated by variability across individuals, comorbid conditions, and methodological differences in biomarker measurement. Consequently, while imaging and biomarker advances are invaluable in advancing mechanistic understanding, their integration into everyday diagnostic frameworks for NPS remains an area for future development.

### 8.3. Neuropsychological testing and its role in NPS

Neuropsychological testing continues to serve as a cornerstone in the diagnosis of cognitive impairments associated with AD and MCI. These assessments provide structured evaluations of memory, attention, language, and executive function, which remain central to the clinical characterization of cognitive decline. However, the application of neuropsychological testing for the diagnosis of NPS has been comparatively limited. While such testing is not traditionally designed to capture affective or behavioral disturbances, it can nonetheless offer valuable insights into the cognitive substrates that contribute to the manifestation of NPS. Neuropsychological profiles can help clinicians distinguish symptoms that arise from neurodegenerative processes and from those associated with primary psychiatric disorders, thereby improving the diagnostic clarity [14],[61].

In recent years, increasing attention has been directed toward adapting standardized neuropsychological instruments to evaluate domains closely tied to NPS, including emotional regulation, executive function, and social cognition. Early deficits in these domains are frequently observed in AD and MCI and are strongly linked to the emergence of behavioral and affective symptoms. For instance, tasks that measure emotion recognition or cognitive flexibility can identify subtle impairments that predispose individuals to depressive features, irritability, or apathy [62],[63]. Such adaptations extend the utility of neuropsychological testing beyond traditional cognitive outcomes and offer a more integrative framework for understanding the interplay between cognitive decline and neuropsychiatric manifestations [63]. Nevertheless, further research is required to validate these approaches and to establish their reliability across diverse populations and clinical settings.

In evaluating the role of neuropsychological testing in the diagnosis of NPS, it becomes evident that such testing, while indispensable for characterizing cognitive decline, remains a limited instrument for capturing the full scope of the behavioral and psychiatric manifestations of dementia [62],[63]. A more holistic approach would integrate neuropsychological findings with neuroimaging, biomarker analyses, and caregiver-based assessments to provide a multidimensional picture of symptomatology. This integration is especially important given the growing recognition that NPS often emerge prior to, or alongside, measurable cognitive decline and may serve as early indicators of disease progression [14]. Future directions should prioritize the development of ecologically valid, culturally sensitive, and longitudinally reliable assessment tools capable of capturing the dynamic interplay between cognition, behavior, and environment.

## 9. Emerging trends in diagnostics

### 9.1. Digital and remote monitoring

With the advent of wearable devices and digital tools, the potential for remote monitoring of NPS in AD and MCI is expanding. Devices that track sleep patterns, physical activity, or even speech patterns can provide objective data on behavioral changes over time, thus offering valuable insights that are often overlooked in traditional clinical settings [64].

For example, smart home technologies and mobile applications that monitor daily functioning can offer real-time information on agitation, disorientation, or social withdrawal. These tools hold promises for enhancing diagnostic accuracy, particularly when caregivers cannot consistently report symptoms [64].

### 9.2. Artificial intelligence and machine learning

Artificial intelligence (AI) and machine learning are being increasingly explored for their potential to diagnose NPS. By analyzing large datasets, AI can identify patterns in clinical, imaging, and genetic data that might be missed by human clinicians. These technologies may improve early diagnoses, enable more personalized treatment plans, and assist in the differentiation of NPS caused by Alzheimer's pathology versus other conditions.

## 10. The link between cognitive decline, dementia, and psychiatric disorders

Neuropsychiatric and cognitive symptoms frequently coexist in the prodromal stages of dementia. Cognitive deficits, especially in memory and executive function, are hallmark features of AD and MCI. However, growing evidence suggests that mood and behavioral changes often predate or parallel these deficits.

In both AD and MCI, NPS are associated with aberrant neurocircuitry involving the limbic system, prefrontal cortex, and medial temporal structures [6],[33]. Functional MRI and PET imaging demonstrate early disruption in fronto-limbic circuits networks implicated in emotion regulation and executive function. Such disruptions can manifest as mood lability, increased irritability, or decreased motivation.

Psychiatric disorders, such as late-life depression and generalized anxiety disorder, not only share symptomatic overlaps with early dementia but also represent potential risk factors or prodromes [6],[33]. For example, major depressive disorders in older adults have been associated with an increased amyloid burden and hippocampal atrophy, both of which are biomarkers of preclinical AD. This complicates the differential diagnosis and necessitates a nuanced understanding of shared pathophysiology.

## 11. Defining features of dementia and psychiatric disorders

### 11.1. Dementia

Dementia is widely recognized as a clinical syndrome characterized by progressive decline across multiple cognitive domains, including memory, language, visuospatial processing, and executive functioning, to a degree that interferes with independent daily activities [55],[65]. Among the various etiologies, AD is the most prevalent, accounting for an estimated 60 to 80 percent of dementia cases worldwide [66]. The burden of AD is profound, not only because of its high prevalence but also due to its significant impact on patients, caregivers, and healthcare systems, making it a major public health concern.

The earliest clinical manifestations of AD typically involve subtle episodic memory impairments, often accompanied by difficulties in attention, problem-solving, and executive control [67]. As the disease progresses, these deficits extend into additional cognitive and behavioral domains, ultimately leading to severe functional dependence. Neuropathologically, AD is defined by the accumulation of extracellular amyloid-beta (Aβ) plaques and the presence of intracellular neurofibrillary tangles composed of hyperphosphorylated tau protein, which together drive neurodegeneration and synaptic dysfunction [68]. These hallmark features provide the foundation for current biomarker-based diagnostic criteria, which increasingly emphasize the biological underpinnings of AD over clinical presentation alone [54].

MCI is conceptualized as a transitional stage between the cognitive changes associated with normal aging and the more severe deficits observed in dementia. Individuals with MCI demonstrate measurable impairments in one or more cognitive domains, most often memory, yet retain relative independence in functional activities [69]. While not all individuals with MCI progress to dementia, the condition is considered a significant risk state, particularly when associated with biomarker evidence of amyloid and tau pathology. Thus, the characterization of MCI plays a critical role in the early detection of AD and in the implementation of timely therapeutic and preventive interventions [70].

### 11.2. Psychiatric disorders and overlap with dementia

Psychiatric disorders such as depression, anxiety, psychosis, and bipolar disorder are primarily characterized by the dysregulation of affect, behavior, and cognition, often in the absence of overt structural brain pathology. In older adults, these conditions frequently co-occur and may complicate or obscure the early recognition of dementia. One example is pseudodementia, a reversible cognitive impairment secondary to depression, which shares many clinical similarities with early AD. However, unlike dementia, pseudodementia typically improves with an appropriate treatment of the underlying mood disorder, thus underscoring the importance of careful diagnostic evaluation [71],[72].

Although psychiatric symptoms in the general population are often episodic and responsive to therapeutic intervention, those that occur in the context of AD or MCI tend to follow a chronic and refractory course. These symptoms are not merely functional disturbances but are biologically anchored in the processes of neurodegeneration, with underlying structural and molecular pathology contributing to their persistence [73].

### 11.3. Neuropsychiatric phenomena in early-stage AD and MCI

NPS are highly prevalent in the early stages of AD and MCI and tend to cluster into recognizable domains. Among the most common are the following: apathy, which affects between 20 and 70 percent of individuals and manifests as diminished motivation and reduced goal-directed behavior; depression, which is observed in 30 to 50 percent of cases and is characterized by pervasive low mood, anhedonia, and cognitive slowing; anxiety, affecting approximately 25 to 50 percent of patients and presenting as excessive worry and fearfulness often accompanied by somatic symptoms; irritability and agitation, seen in 20 to 40 percent of patients and reflects heightened emotional reactivity; and sleep disturbances, reported in 30 to 60 percent of cases, including insomnia, fragmented sleep, and circadian rhythm disruption. Less common, though clinically significant, are symptoms such as delusions, hallucinations, and disinhibition. While these typically emerge later in the disease trajectory, they may also appear early in atypical or rapidly progressing presentations [21],[74]. The underlying pathophysiology of NPS in early-stage AD and MCI reflects multiple converging mechanisms.

Neurodegeneration, particularly medial temporal lobe atrophy and frontal hypometabolism, disrupts the circuits involved in mood and behavioral regulation. Neurotransmitter imbalances, including deficits in serotonergic, dopaminergic, and glutamatergic signaling, are also implicated in the emergence of depressive and psychotic symptoms. Chronic neuroinflammation, mediated by microglial activation and cytokine release, further alters the neurochemical milieu and contributes to symptom expression. In addition, genetic susceptibility plays a role, with carriers of the APOE ε4 allele demonstrating elevated risk not only for cognitive impairment but also for NPS [35],[73].

### 11.4. Clinical impact of NPS in early-stage cognitive disorders

The burden of NPS extends beyond patients to caregivers, who frequently experience heightened stress, depression, and anxiety [74]. Caregiver burden associated with NPS often precipitates premature institutionalization, as behavioral disturbances such as agitation, aggression, or psychosis are consistently reported as the most common reasons for residential placements, thereby surpassing memory loss in predictive value [29].

### 11.5. Treatments perspectives

The management of NPS in early-stage AD and MCI is multifaceted, with increasing emphasis on individualized, multimodal approaches. Non-pharmacological interventions are widely regarded as first-line strategies given their more favorable safety profile and the risks associated with pharmacological treatments in older adults. Cognitive-behavioral therapy (CBT) has demonstrated modest but meaningful benefits in reducing anxiety and depressive symptoms, while behavioral activation strategies specifically target apathy by encouraging re-engagement in structured and rewarding activities [75]. Caregiver education and psychosocial support interventions further improve outcomes by equipping families with strategies to manage agitation, mood fluctuations, and environmental triggers. Environmental modifications, such as the use of structured routines, a reduction of overstimulation, and a provision of orientation aids, have also been shown to mitigate confusion and agitation in early dementia [76].

Pharmacological treatment remains an important adjunct in cases where non-pharmacological approaches prove insufficient or where symptoms are severe, persistent, or pose safety risks. Antidepressant medications that modulate neurotransmitter systems, particularly selective serotonin reuptake inhibitors (SSRIs) such as sertraline and citalopram, have shown some efficacy in alleviating depressive symptoms in dementia, though their overall benefit is modest and inconsistent across studies [77],[78]. Antipsychotic agents, including risperidone and olanzapine, are sometimes used to manage severe agitation, aggression, or psychosis. However, their use is limited by concerns regarding increased risks of cerebrovascular events, sedation, and mortality in elderly populations with dementia [79]. For this reason, clinical guidelines emphasize the cautious, time-limited use of antipsychotics, with close monitoring and regular attempts at withdrawal when feasible.

Cholinesterase inhibitors (e.g., donepezil, rivastigmine, galantamine) and memantine, which are primarily utilized for cognitive symptoms, have demonstrated secondary benefits for certain behavioral and affective disturbances, including apathy and agitation [80]. Therefore, these agents may play a dual role in addressing both cognitive and neuropsychiatric domains, though the effect sizes remain small. Importantly, pharmacological interventions should always be implemented in conjunction with, rather than as substitutes for, non-pharmacological strategies, as the latter provide a foundation for safer and more sustainable symptom management.

### 11.6. Critical considerations

The choice of treatment must balance efficacy with safety, particularly given the increased vulnerability of older adults with cognitive impairments to adverse drug effects. While pharmacological interventions remain necessary in selected cases, an overreliance on them can expose patients to iatrogenic risks without producing substantial long-term benefits. Therefore, future research should focus on personalized, biomarker-informed treatment strategies that tailor interventions to the underlying neurobiological mechanisms of NPS. Moreover, integrated care models that combine pharmacological treatments, psychosocial interventions, caregiver support, and environmental modifications are likely to yield the most meaningful improvements in the patients QoL and the caregiver's well-being.

## 12. Future directions and research needs

Understanding the biological foundation of NPS in AD and MCI remains a critical priority to advance early detection and intervention. Although progress has been made in delineating the prevalence and clinical implications of NPS, major gaps remain in understanding the underlying mechanisms that drive their emergence and in translating this knowledge into effective treatment strategies. A central focus of future research should be the detection of early predictive biomarkers that forecast disease progression, and that can differentiate between symptom profiles and disease trajectories. For example, functional neuroimaging has begun to reveal associations between apathy, depression, and altered activity in fronto-limbic circuits, but further validation is needed to establish these findings as reliable diagnostic markers [56],[58].

Given the late life emergence of neuropsychiatric symptoms and risk of dementia in cognitively unimpaired older adults [81], a longitudinal study investigated how the emergence of new, significant NPS in cognitively unimpaired older adults is associated with subsequent cognitive decline and conversion to dementia. The study tracked the temporal evolution of NPS in relation to cognitive decline as essential to clarifying the prognostic value of these symptoms [81]. Evidence suggests that the presence of apathy and depression in MCI significantly increases the risk of conversion to AD, yet the mechanisms underlying this association remain poorly understood [14]. Carefully designed, large-scale, prospective studies would help to disentangle whether NPS serve as early indicators of neurodegeneration or as accelerants of disease progression, thereby informing both diagnostic frameworks and therapeutic strategies.

Equally important is the development of emphasizing the tailoring of interventions to the specific clinical and psychosocial profiles of each patient. For example, future research should focus on developing personalized treatment approaches based on neuropsychiatric symptom profiles. These treatment protocols should integrate neurobiological, cognitive, and psychosocial factors. Recognizing that NPS cluster into distinct symptom domains such as mood disturbances, apathy, and agitation offers an opportunity to tailor interventions to specific behavioral phenotypes and their underlying pathology. Precision medicine approaches that incorporate biomarker data, neuroimaging profiles, and genetic risk factors could allow for more targeted interventions that simultaneously address both cognitive decline and behavioral manifestations [80].

The integration of digital health tools represents another promising avenue for innovation. Remote symptom monitoring platforms, wearable devices, and mobile applications have the potential to provide real-time assessments of mood, behavior, and sleep, thus offering clinicians and caregivers a continuous stream of data to guide treatment adjustments [82]. These tools not only improve access to care but also empower caregivers with resources for education and support, thus mitigating caregiver burden while enhancing early detection of behavioral changes.

It is important to acknowledge that translational models that bridge molecular pathology and behavioral phenotypes are urgently needed to accelerate drug development. Animal models and human biomarker studies which link amyloid and tau pathology, neuroinflammation, and neurotransmitter dysregulation to specific neuropsychiatric presentations could inform the development of novel pharmacological interventions [59],[60]. Such efforts would contribute to a mechanistic framework in which NPS are understood not as peripheral symptoms but as integral components of disease biology. By addressing these priorities, future research has the potential to transform NPS from a source of diagnostic uncertainty into a pathway for early intervention, improved patient outcomes, and a reduced caregiver burden.

## 13. Discussion

The synthesis of the selected literature supports a fundamental reorientation in how NPS are conceptualized in the context of early AD and MCI. Rather than constituting secondary, reactive, or “late-stage” manifestations, NPS emerge as core clinical features that are biologically anchored in early neurodegenerative change and exert independent effects on prognosis, quality of life, and healthcare utilization. At the same time, a critical appraisal of the evidence reveals substantial methodological limitations, heterogeneity in definitions and measures, and persistent inequities that constrain the translation of current knowledge into practice.

### 13.1. NPS as core features of early neurodegeneration: Integration and caveats

The literature reviewed consistently demonstrates that NPS-particularly apathy, depression, anxiety, irritability, agitation, and sleep disturbance-are highly prevalent in early-stage AD and MCI and often precede overt cognitive deficits. This supports a shift away from viewing these symptoms as mere psychological reactions to cognitive decline and toward recognizing them as early and integral manifestations of the disease process. Structural and functional neuroimaging studies linking NPS to medial temporal, limbic, and frontal-parietal circuit abnormalities, together with evidence of neurotransmitter dysregulation and genetic vulnerability, reinforce this biologically grounded interpretation.

However, a critical examination reveals several caveats. First, many of the included studies are cross-sectional or rely on relatively short follow-up periods, limiting causal inference regarding whether NPS signal underlying pathology, accelerate disease progression, or both. Second, operationalization of NPS considerably varies across studies (e.g., different instruments, cut-points, and symptom clusters), thus making direct comparisons and meta-analytic syntheses challenging. Third, there is a risk of circular reasoning: once individuals are identified as having or being at risk for AD, NPS may be more readily attributed to neurodegeneration, whereas similar symptoms in other contexts are labeled as primary psychiatric disorders. This diagnostic bias may inflate the perceived specificity of NPS for early AD and MCI.

Despite these limitations, the weight of converging evidence supports the view that at least some NPS-especially apathy and depression are not epiphenomenal but carry independent prognostic significance. The critical task for future work is to disentangle which NPS (or which combinations and trajectories) are most strongly and specifically associated with underlying AD pathology, and under what conditions they are more likely to reflect psychosocial or primary psychiatric processes.

### 13.2. Prognostic significance and disease modifying potential: Strengths and uncertainties

A key contribution of this narrative review is the emphasis on NPS as predictors and modifiers of disease trajectory. Longitudinal cohorts indicate that the presence of NPS in MCI, particularly apathy and depression, is associated with a higher likelihood of progression to AD and a faster rate of cognitive and functional decline. These findings are clinically meaningful, as they suggest that NPS could be incorporated into risk stratification models and used to identify individuals who may benefit most from intensified monitoring or early interventions.

However, the prognostic data requires cautious interpretations. Many studies do not fully adjust for potential confounders such as vascular comorbidities, sleep disorders, medication effects, or pre-existing psychiatric history. Reverse causality is also a concern: individuals in whom neurodegeneration is already more advanced may be more likely to exhibit NPS, thus creating the appearance of predictive value when NPS may simply co-occur with a more aggressive pathology. Moreover, the effect sizes are often modest, and the incremental prognostic gain of adding NPS to established biomarkers (e.g., amyloid/tau imaging, CSF markers) remains unclear.

Critically, the literature rarely addresses whether an active treatment of NPS alters the trajectory of cognitive decline or delays conversion from MCI to dementia. The assumption that NPS represent modifiable risk factors rather than merely markers of disease severity remains largely untested. This is a major gap: without intervention studies that target NPS and track downstream cognitive and functional outcomes, we cannot determine whether NPS are viable therapeutic levers or primarily indicators of underlying disease burden.

### 13.3. Diagnostic complexity: Toward integration, not oversimplification

The review appropriately underscores that NPS can both illuminate and obscure the diagnostic process. On one hand, new-onset or atypical NPS in later life may serve as early clinical clues of neurodegeneration, particularly when coupled with subtle cognitive changes. On the other hand, substantial symptoms overlap with primary psychiatric disorders (e.g., late-life depression, anxiety, bipolar disorder), which complicates differential diagnoses.

### 13.4. In critically appraising the diagnostic literature, several tensions emerge

Over-attribution vs. under-recognition: There is a dual risk of either attributing all late-life NPS to “incipient dementia” or, conversely, dismissing early behavioral change as “just depression” or normal aging. Both errors have consequences by either over-medicalizing psychiatric symptoms or delaying the diagnosis of a neurodegenerative disorder.

Instrument limitations: Assessment tools such as the NPI, GDS, and CSDD are widely used and valuable but have inherent constraints. The heavy reliance on caregiver reports is vulnerable to informant burden, cultural interpretations, and recall bias. Self-report measures lose validity as cognitive impairment progresses. Very few instruments were designed to distinguish NPS arising from early AD/MCI from those due to primary psychiatric conditions.

Partial integration of biomarkers and imaging: While neuroimaging and biomarkers are increasingly used to stage AD, they are not yet systematically incorporated into diagnostic workflows specifically for NPS. Existing studies often come from specialized research centers, use small samples, and lack standardized thresholds for linking biomarker abnormalities to distinct NPS profiles.

Thus, although this review persuasively argues for integrating NPS into early diagnostic frameworks, a critical perspective suggests that current tools and criteria remain ill-equipped for this task. There is a need for diagnostic algorithms that explicitly incorporate NPS alongside cognitive, biomarker, and contextual data, as well as for training clinicians to appreciate when NPS should raise suspicion for underlying neurodegeneration rather than be treated solely as primary psychiatric entities.

### 13.5. Quality of life, caregiver burden, and system-level consequences: Beyond symptom counts

The reviewed literature convincingly demonstrates that NPS exert profound and early effects on the QoL for both the patients and the caregivers. Apathy, mood disturbance, irritability, disinhibition, and sleep disruption undermine engagement in meaningful activities, strain relationships, and precipitate functional decline even when cognitive deficits are relatively mild. For caregivers, NPS are more predictive of burnout and institutionalization than memory loss alone.

Critically, however, QoL and burden are often treated as downstream outcomes rather than central endpoints in their own right. Many studies prioritize cognitive endpoints or conversion to dementia, thereby relegating QoL metrics to secondary status. Furthermore, the measurement of QoL and caregiver burden is itself subject to cultural, socioeconomic, and gender-related influences that may not be fully captured by standardized scales. For example, behaviors labeled as “agitation” or “disinhibition” may be perceived and tolerated differently across cultural contexts, shaping both reported burden and decisions about institutionalization.

From a systems perspective, the review highlights substantial economic costs associated with NPS, including increased psychotropic use, hospitalizations, and long-term care placement. Yet there is relatively little cost-effectiveness research which compares early, proactive management of NPS with usual care. A critical reading suggests that while the burden of NPS is well documented, the field has not yet generated robust evidence on which specific interventions, delivered at which time points, produce meaningful and cost-effective improvements in the QoL and caregiver outcomes.

### 13.6. Equity, diversity, and generalizability: Who is seen, who is missed

One of the most salient strengths of this review is its explicit attention to racial, ethnic, gender, socioeconomic, and cultural disparities in NPS expression and care. The literature indicates that minoritized and socioeconomically disadvantaged groups may experience different patterns of symptoms (e.g., higher reported rates of psychosis or agitation), face greater barriers to diagnosis and treatment, and bear disproportionate caregiver burden.

However, the critical analysis must also acknowledge that the existing evidence base on NPS is itself skewed. Many of the longitudinal cohorts and clinical trials that inform our current understanding are disproportionately composed of non-Hispanic White, highly educated, urban-dwelling participants. Diverse populations are often underrepresented, enrolled in small numbers, or included without adequate subgroup analyses. This raises important questions about the external validity of findings such as the following: are reported prevalence rates and symptom profiles truly reflective of underlying biology, or do they partly reflect cultural norms, diagnostic bias, and differential access to care?; do neuroimaging and biomarker correlates of NPS generalize across racial/ethnic groups, or are there population-specific patterns that have not yet been adequately characterized?; and how do structural factors such as health system inequities, stigma, and linguistic barriers-shape the observed distribution of NPS, caregiver burden, and institutionalization?

By drawing attention to these questions, this review contributes not just descriptive knowledge of disparities but also a critique of how current research practices may perpetuate them. It argues, implicitly and explicitly, for a shift toward inclusive recruitment strategies, culturally validated assessment tools, and community-engaged research approaches that can generate findings relevant to the populations most affected by dementia.

### 13.7. Treatment perspectives: Promise of multimodal, personalized care amid limited evidence

The treatment literature, as synthesized here, points toward a broad consensus: non-pharmacological interventions should be first-line for most NPS in early AD and MCI, with pharmacological treatments reserved for severe or refractory symptoms. Behavioral activation, environmental modification, structured routines, caregiver training, and psychosocial interventions consistently show small-to-moderate benefits with fewer risks compared to psychotropic medications. Pharmacological agents, including SSRIs, antipsychotics, and cognitive enhancers, offer limited and often inconsistent benefits and carry well-known safety concerns in older adults.

### 13.8. From a critical standpoint, several issues warrant emphasis

Evidence quality: Many non-pharmacological studies are limited by small sample sizes, heterogeneous interventions, a lack of blinding, and short follow-ups. Heterogeneity in interventions makes it difficult to identify which specific components are most effective.

Implementation gap: Even when effective non-pharmacological strategies are identified, they are seldom systematically implemented in routine care due to resource constraints, limited caregiver support, workforce shortages, and a lack of reimbursement structures.

Overreliance on psychotropics: In practice, psychotropic medications remain heavily used, often in ways that exceed the guideline recommendations. This reflects not only clinical habits, but also structural realities and non-pharmacological interventions which require time, training, and infrastructure that many systems lack.

The discussion of emerging approaches, such as neuromodulation, biomarker-informed treatments, digital monitoring, and AI-driven analytics, offers an important forward-looking dimension. Nonetheless, these technologies must be critically appraised with regard to feasibility, equity, and real-world impact. Many are currently confined to research settings or high-resource environments, risk widening existing disparities in access to high-quality dementia care, and have not yet demonstrated long-term effectiveness on patient-centered outcomes.

### 13.9. Methodological constraints and the role of narrative synthesis

The decision to employ a structured narrative review, rather than a formal meta-analysis, reflects the heterogeneity of the available literature. This design allows for integration across domains—neurobiology, diagnostics, disparities, and clinical management—that are often studied in isolation. However, it also introduces potential biases, including the selective emphasis and unequal weighting of evidence.

Critically, the narrative approach cannot fully resolve issues such as publication bias (e.g., underreporting of negative or null findings), variability in NPS definitions, and inconsistent adjustments for confounding variables. The strength of this review lies in its conceptual integration and identification of cross-cutting themes; its limitations lie in its inability to quantify the effect sizes or definitively rank interventions and predictors. Recognizing these constraints is essential to appropriately situate its conclusions within the broader evidence base.

### 13.10. Overall significance and direction for the field

Despite the limitations of the underlying literature, this narrative review makes a substantive contribution by reframing NPS as early, biologically grounded, and prognostically meaningful features of AD and MCI, rather than peripheral complications. It critically examines diagnostic and therapeutic practices, highlights the risks of both under-recognition and over-attribution, and underscores the need for integrated, biomarker-informed, and culturally sensitive frameworks.

Foregrounding equity and diversity, calling attention to who is and is not represented in current research, and how structural factors shape the observed landscape of NPS and their management.

Priorities for future work include the following: longitudinal, diverse cohorts; mechanistic studies linking biomarkers to specific NPS trajectories; and rigorous trials of multimodal, personalized interventions that explicitly incorporate caregiver and QoL outcomes.

In summary, this review positions NPS as a critical lens through which we understand and intervene in early AD and MCI. It argues that meaningful progress in dementia care will require moving beyond a cognition-centric model toward one that fully integrates neuropsychiatric, biological, social, and cultural dimensions. Doing so holds the potential to not only refine early detection and prognostication but also to develop more humane, equitable, and effective models of care for individuals and families navigating the earliest stages of neurodegenerative disease.

## 14. Conclusions

NPS are not peripheral but central to the clinical expression and trajectory of AD and MCI. They emerge early, predict poorer outcomes, and profoundly affect patients and caregivers. Despite their high prevalence, NPS remain under-recognized and undertreated.

NPS are a critical factor in the progression of AD and MCI significantly impacting not only the patients but also their caregivers. Symptoms such as depression, apathy, anxiety, and aggression can substantially increase the caregiver burden, thus leading to emotional, physical, and financial strain. As these symptoms exacerbate the challenges of caregiving, they often result in earlier nursing home placements, which, while necessary for some, compounds the emotional distress of caregivers.

The treatment of NPS remains a complex issue, with current pharmacological treatments often offering limited relief and posing potential risks. Non-pharmacological interventions show promise, but their application is often limited by caregiver resources and availability. Therefore, addressing NPS through comprehensive, individualized care plans that incorporate both pharmacological and non-pharmacological strategies is essential. Furthermore, ongoing research into more effective treatments and interventions is crucial to alleviate the burden on caregivers and improve outcomes for both the patients and their families.

The treatment of NPS in early AD and MCI is multifaceted, thereby requiring a balanced approach that incorporates both pharmacological and non-pharmacological interventions. Although pharmacological treatments are essential, particularly for managing more severe symptoms, non-pharmacological strategies such as cognitive behavioral therapy, caregiver support, and environmental modifications play an equally important role in improving patient outcomes and alleviating caregiver burden. As research progresses, emerging therapies that target underlying neurobiological mechanisms may provide additional tools to manage these debilitating symptoms more effectively and safely.

Effective management requires an integrated approach that combines behavioral interventions, caregiver support, and targeted pharmacological strategies. Advancing our understanding of the neurobiological and clinical underpinnings of NPS will be pivotal to develop comprehensive care models for individuals in the early stages of cognitive decline.

## Use of AI tools declaration

The authors declare they have not used Artificial Intelligence (AI) tools in the creation of this article.
